# Long-term data from field erosion plot studies in eastern Austria

**DOI:** 10.1016/j.dib.2020.105810

**Published:** 2020-06-03

**Authors:** Andreas Klik, Josef Rosner

**Affiliations:** a University of Natural Resources and Life Sciences Vienna, Department of Water, Atmosphere and Environment, Institute for Soil Physics and Rural Water Management, Vienna, Austria; b Office of the Lower Austrian Provincial Government, Division of Culture, Science and Education, St. Pölten, Austria

**Keywords:** Soil erosion, surface runoff, conservation tillage, erosion plots, Austria

## Abstract

Soil erosion and runoff data are collected at three sites in eastern Austria using field erosion plots. Observed treatments include 1) conventional tillage with plough (CT), 2) mulch tillage with winter cover crop (MT), and 3) no-till with winter cover crop (NT). Data cover a time span from 1994 to 2018. They include data about surface runoff, soil loss, nitrogen, phosphorus and soil organic carbon losses as well as cop yields associated with the erosion processes. Interpretation of the data will be found in “Long-term experience with conservation tillage practices in Austria: impacts on soil erosion processes” [1].

**Specifications Table** [Every section of this table is mandatory. Please enter information in the right-hand column]

**Table:** 

Subject	Soil science
Specific subject area	Soil and water conservation
Type of data	Tables
How data were acquired	Long-term field experiments with different tillage practices were conducted at three sites in eastern Austria.
Data format	Raw Analyzed
Parameters for data collection	From 1994 to 2018 field experiments were conducted to investigate the impact of different tillage practices on soil erosion processes as well as on crop yield. Measurements were carried out in the eastern part of Austria in Mistelbach, Pixendorf and Pyhra. Runoff, soil and nutrient loss data were obtained using field erosion plots of 45 and 60 m² [2]. Three tillage practices were investigated including conventional tillage, mulch tillage and no-till practice. Runoff and sediment samples were brought to the laboratory after each erosion event and further physically and chemically analysed. Crop yield was determined from 45 m² large plots with replications. For each tillage practice and for each year data include planted crop, corresponding crop yields and yield relative to CT, annual precipitation, surface runoff, soil loss, nitrogen, phosphorus and soil organic carbon losses. Data for Mistelbach are included in Table 1, data for Pixendorf and Pyhra in Tables 2 and 3, respectively.
Description of data collection	Runoff and soil loss data were obtained using field erosion plots of 45 and 60 m² [2]. Runoff and sediment samples were brought to the laboratory after each erosion event and further physically and chemically analysed. Crop yield was determined from 45 m² large plots with replications.
Data source location	City/Town/Region: Mistelbach, Pixendorf, Pyhra, Country: Austria, Latitude and longitude (and GPS coordinates) for collected samples/data:], Mistelbach 48° 34' 58'' N 16° 35'09'' E, Pixendorf 48° 16'56'' N 15° 59' 01'' E, Pyhra 48° 08'58'' N 15° 42' 12'' E
Data accessibility	Repository name: zenodo, Data identification number: 10.5281/zenodo.3660940, Direct URL to data: https://zenodo.org/record/3660940#.Xqguj6gzZPY
Related research article	Author's name: Andreas Klik and Josef Rosner, Title: Long-term experience with conservation tillage practices in Austria: impacts on soil erosion processes, Journal: Soil and Tillage Research, DOI: 10.1016/j.still.2020.104669

**Value of the Data**

• Long-term data about impact of conservation tillage practices on soil erosion processes are very sparse.
• The long-term database is useful for stakeholders and decision makers to adapt existing and develop future soil protection strategies
• The data can be used to calibrate and validate soil erosion simulation models for central European conditions

## 1 Data

The data include measured data for three different soil tillage practices from three experimental sites in eastern Austria. The Tables 1 includes for each year the period of operation, the planted crop, the annual precipitation, the surface runoff in mm, the soil loss in t/ha and the surface losses of total nitrogen, total phosphorus and soil organic carbon in kg/ha in Mistelbach. Tables 2 and 2 include corresponding data for Pixendorf and Pyhra. Crop yields were determined from 45 m² large plots with one or two replications. Yields in kg/ha are not available for all years, but relative yield in % are.

## 2 Experimental Design, Materials, and Methods

### 2.1 Study area and treatments

The experiments were conducted at three sites in eastern Austria: in Mistelbach, Pixendorf and Pyhra

The soils in Mistelbach and Pyhra are classified as Typic Argiudolls while the soil in Pixendorf is an Entic Hapludoll. Soil textures range from silt loam to loam. Average annual rainfall (1994-2018) at the sites amounts from 621 to 916 mm with average annual air temperatures between 9.4 and 10.4°C.

Following soil tillage treatments were investigated: (1) conventional tillage system with ploughing in fall (CT), (2) mulch tillage with cover crops during winter (MT) and (3) no-till with cover crops during winter (NT). The study design was a randomized block and each treatment was replicated twice.

### 2.2 Erosion plots and field measurements

Each treatment was equipped with one field runoff plot. The experimental plots were 6 m wide and – depending on site conditions - between 40 and 80 m long.

The study design consisted of 3 (Mistelbach) and 4 m wide (Pixendorf, Pyhra) and 15 m long runoff plots for each management variation [2]. Each plot was bordered by stainless steel metal sheets. At the lower end of the plot surface runoff and soil loss were collected in a trough and then diverted by a 100-mm PVC pipe to an Automated Erosion Wheel (AEW) [3]. The design of this AEW is similar to a tipping bucket and consists of four equal sections of five liters resulting in a resolution of each tip of 0.08 mm for 60 m² plots. A magnetic sensor system was used for continuous runoff measurement.

Soil-water-suspension was divided by an adapted multi-tube divisor taking 3.3% of the sample that is collected in a 60 L collection tank. After each erosive storm the collection tank was emptied and the runoff sample was brought to the laboratory, weighed and dried until constant mass was achieved to determine sediment concentration. Based on the continuously measured runoff data from the data logging system and the sediment concentration the amount of soil loss from the plots was calculated for each erosive event.

Throughout the investigation period soil erosion, surface runoff and nutrient and carbon losses and partly also pesticide losses due to erosion processes were determined for all sites and tillage systems. Immediately after planting/seeding of summer crops the erosion plots were installed and then operated until harvest. After the harvest the equipment was removed. No measurements were performed during winter due to limited accessibility and frost damages to the equipment. At each site an automatic tipping bucket rain gauge was placed to measure rainfall in 5-min intervals.

Crop yield was determined from each treatment with three replications.

### 2.3 Physical and chemical analyses of water and sediment samples

Total nitrogen and total carbon in sediment samples was analysed by dry combustion [4] using a C/N Analyzer (Vario Max CN, Elementar). Soil organic carbon content was obtained by subtracting inorganic carbon content measured volumetrically by the Scheibler method with a Calcimeter [5]. Total phosphorus of the sediment was determined using a UV/VIS spectral photometer (DU-640 Beckmann) [6].

Pesticides were extracted from water by solid phase extraction and from sediments using distilled water or organic solvent [7]. After shaking for several hours and centrifugation, the sample passed through a solid phase extraction. After evaporation of the extract the pesticide residues were redissolved in another solvent and analysed with high performance liquid chromatography (HPLC). Year and period of operation of long-term runoff plots, amount of surface runoff, soil loss, nitrogen and phosphorus loss as well as absolute and relative crop yields for Mistelbach are displayed in Table 1, for Pixendorf in Table 2 and for Pyhra in Table 3.

## Declaration of Competing Interest

The authors declare that they have no known competing financial interests or personal relationships that could have appeared to influence the work reported in this paper.

## Appendix Supplementary materials

Image, application 1
